# Three Centuries of Synchronous Forest Defoliator Outbreaks in Western North America

**DOI:** 10.1371/journal.pone.0164737

**Published:** 2016-10-13

**Authors:** Aquila Flower

**Affiliations:** Department of Environmental Studies, Huxley College of the Environment, Western Washington University, Bellingham, Washington, 98225, United States of America; Natural Resources Canada, CANADA

## Abstract

Insect outbreaks often occur synchronously across large spatial scales, but the long-term temporal stability of the phenomenon and the mechanisms behind it are not well understood. In this study, I use a widespread lepidopteran defoliator native to western North America—the western spruce budworm—as a case study to explore patterns of and potential causes for synchronous population fluctuations. Analyses of synchrony are typically severely limited by the short historical records available for many species. To overcome this limitation, I compiled multi-century dendrochronological reconstructions of western spruce budworm outbreaks from across much of the species’ range. This allowed me to analyze synchrony at a sub-continental spatial scale over the last three centuries. I found statistically significant synchrony among regional outbreak records up to 2,000 km apart and identified numerous outbreak periods that occurred synchronously across much of the species’ range. I quantified spatial and temporal associations between climate and synchronous outbreak periods using paleoclimate reconstructions. The spatial patterns of outbreak histories and climate records were remarkably similar, with higher similarity in outbreak histories apparent between regions with more similar climate conditions. Synchronous outbreaks typically occurred during periods of average or above average moisture availability preceded by periods of low moisture availability. My results suggest that climatic variability has played a key role in synchronizing western spruce budworm population fluctuations in disjunct forests across western North America for at least the last three centuries. Widespread synchrony appears to be a natural part of this species’ population dynamics, though synchronous outbreaks have occurred more frequently during the 20^th^ century than during prior centuries. This study uses a novel combination of statistical methods and dendrochronological data to provide analyses of this species’ population dynamics with an unprecedented combination of spatial extent and temporal depth.

## Introduction

Numerous studies have identified synchronous fluctuations in spatially disjunct populations of insects (e.g., [1]), mammals (e.g., [2]), fish (e.g., [3]), birds (e.g., [4]), and plants (e.g., [5]). Such spatial synchrony typically decreases with distance, though it may be significant at distances over 1,000 km [2,6]. Quantifying spatial synchrony and identifying the causal mechanisms behind observed patterns of synchrony is crucial for understanding species’ population dynamics, quantifying natural disturbance regimes, designing conservation strategies, aiding efforts to manage outbreaks of “pest” species [7,8], and predicting the likely impacts of future climate change on species with climatically-driven population dynamics. In spite of the plethora of examples of synchrony and the importance of understanding this topic, the exact causal mechanisms behind this phenomenon are not fully understood for many species. Both biotic and abiotic mechanisms have been postulated, but it is often difficult to disentangle the multiple interacting potential causal factors [9,10]. Proposed causal mechanisms fall largely into three categories: first, dispersal of individual organisms from areas of high population density to areas of low population density; second, exogenous abiotic forcing factors such as climatic variability; and, third, trophic interactions with other highly mobile predator or prey species [11]. A problem frequently encountered in analyses of synchrony is the short length of historical records of population fluctuations for many species. This leaves a great deal of uncertainty regarding whether available records are representative of the long-term population dynamics of a species. In this study, I use ecological statistical methods and multi-century dendrochronological records to conduct an analysis of synchrony with an exceptional combination of spatial extent and temporal depth.

I use the western spruce budworm (*Choristoneura occidentalis* Freeman; hereafter referred to as WSB) as a case study for quantifying population synchronization at a sub-continental spatial scale. This species is widely recognized as the most destructive defoliator in western North America and defoliates forests across much of a broad range extending from just north of the US-Mexico border to northern British Columbia [12]. The larvae of this species feed primarily on Douglas-fir (*Pseudotsuga menziesii* (Mirb.) Franco), grand fir (*Abies grandis* (Doug. ex D. Don) Lindl.), and white fir (*Abies concolor* (Gord. and Glend.) Lindl. ex Hildebr.). Defoliation by WSB leads to reduced radial and vertical growth, increased susceptibility to subsequent infestations by other insects and pathogens, and mortality of limbs or entire trees, especially in the case of saplings [13]. Outbreaks commonly last, on average, between nine and 12 years in both the Pacific Northwest [14–16] and the American Southwest [17].

Synchrony of WSB outbreaks across regions encompassing hundreds to tens of thousands of square km is a common feature in western North American forests [15], but little research has focused on identifying, analyzing, or explaining patterns of *inter-regional* synchrony at sub-continental scales. Furthermore, very little is known about the influence of climate on large-scale WSB population dynamics. Understanding synchronous species’ population dynamics is challenging because robust observational records of outbreak events are typically not available prior to the mid-twentieth century in North America [18]. Local historical records of WSB populations therefore often cover only two or three outbreak cycles. Previous analyses of widespread WSB synchrony have thus been limited to the period since around the mid-twentieth century [1].

Long-term records are particularly critical for establishing the natural range of variability of insect outbreaks given the extensive ecological alteration that has occurred over the last century due to land use practices and climate change. Fortunately, dendrochronological methods can be used to reconstruct annually resolved histories of WSB outbreak dates, thus extending our outbreak records by centuries [19,20]. These techniques are based on identification of WSB outbreaks recorded in tree rings as years of reduced radial growth [19]. The effects of defoliation on radial growth can be separated from the effects of climatic variability by comparing the ring-width series from tree species known to serve as hosts for the WSB with ring-width series from other tree species that are not susceptible to WSB outbreaks, provided both species respond to climatic variability in a similar manner [19]. Dendrochronological reconstructions of WSB outbreak history can thus provide the temporal depth needed to analyze WSB population dynamics over numerous outbreak cycles and compare current outbreak characteristics with those from before the onset of fire exclusion, anthropogenic climate change, extensive logging, and other recent ecological alterations.

In this paper, I use previously published dendrochronological records of WSB outbreak dates to assess spatial and temporal patterns of synchrony across western North America over the last three centuries. I compare these records with paleoclimate records of moisture availability to quantify the association between climate and the initiation and cessation of synchronous outbreaks occurring across multiple regions. Specifically, I test 1) how often WSB outbreaks have occurred synchronously over sub-continental spatial scales during the last three centuries, and 2) whether large-scale synchronous WSB outbreaks are associated with spatial and temporal climate patterns. The use of a compiled dataset of dendrochronological records allows me to analyze synchrony over a greater spatial extent (New Mexico to British Columbia) and time period (several centuries) than possible in prior studies.

## Materials and Methods

### Outbreak histories

**Fig 1:**
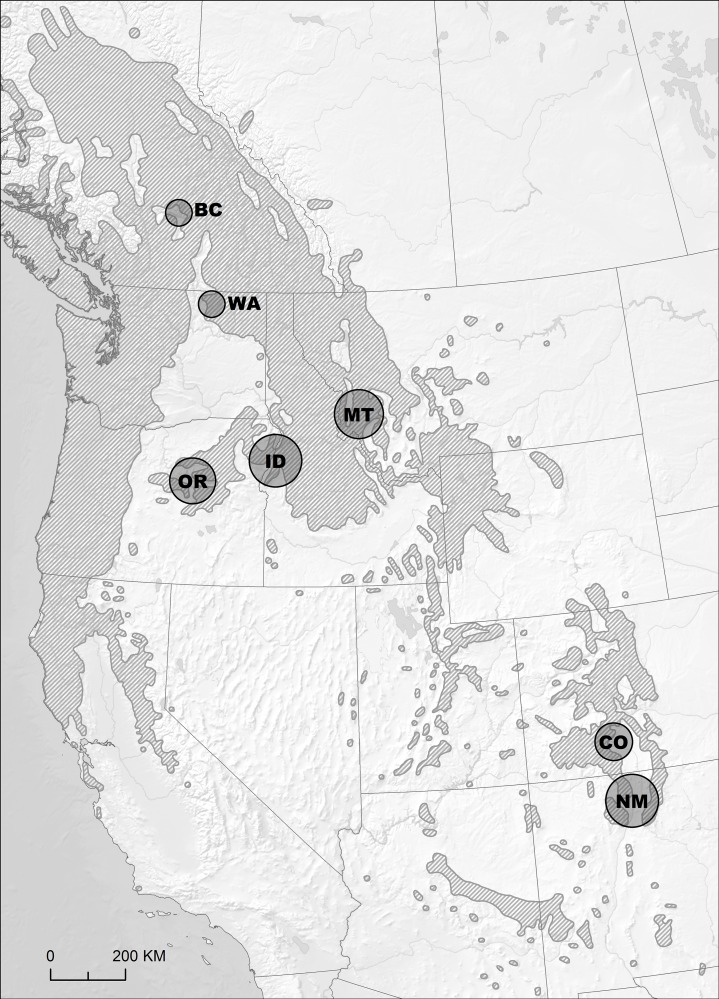
Approximate location and relative size (not to scale) of study regions for the western spruce budworm outbreak reconstructions analyzed in this paper. Shaded area shows the distribution of Douglas-fir (the primary host species for western spruce budworm). Distribution data from Little (1971) [22], base map made with data from Natural Earth.

To assess synchrony of WSB outbreaks over time scales longer than available observational records, I compiled and compared outbreak records from previously published dendrochronological studies (Fig 1) in seven regions: southern British Columbia [14], northeastern Washington [16], eastern Idaho through western Montana [15], northeastern Oregon through western Idaho [15], central Oregon [15], southern Colorado [21], and northern New Mexico [17]. In each region, the original authors reconstructed WSB outbreaks using broadly similar protocols, though each relied on slightly different specific criteria and thresholds for outbreak identification. The general approach to outbreak identification involves statistical comparison of annual ring-width series from “host” tree species known to be susceptible to the WSB with ring-width series from “non-host” tree species that are not typically fed on by the WSB. Non-host chronologies are standardized to have the same mean and variance as the host chronologies and then subtracted from the host tree chronologies, thereby largely removing the climate signal contained in the host trees and isolating the outbreak signal (see [19] for a detailed description of the basic methodological approach). Outbreaks are identified in these “corrected” chronologies by systematically searching for time periods in which the radial growth reduction exceeds both minimum magnitude and minimum duration thresholds in order to highlight periods of relatively long lasting and severe growth reductions. It is difficult to determine the effects of sample collection protocols, standardization techniques, and outbreak identification criteria used in separate studies on their resulting outbreak reconstructions. However, it is clear that extensive familiarity with local outbreak patterns and careful comparison with observational outbreak records is necessary to select appropriate criteria for outbreak identification. I therefore chose to rely on the original authors’ local expertise and use their published definitions of local outbreaks.

Each study included in this paper provided a set of years in which outbreaks occurred within each region. Where possible, I used the original authors’ definition of regional (as opposed to stand-level) outbreaks for their specific study region. Flower et al. (2014) produced stand-level outbreak chronologies at 13 sites across a 600 km transect. For the purpose of this analysis, I converted Flower et al.’s (2014) stand-level outbreak chronologies to regional outbreak records covering spatial extents approximately equal to those covered by the other studies used in this analysis. To do so, I divided the 13 sites into three regions and then calculated the percent of trees recording an outbreak across all sites within each region. I determined the three regions based on how isolated each cluster of sites was from other clusters, as well as the relative strength of the correlation among outbreak time series as reported by Flower et al. (2014). I labeled years in which at least 40% of all trees in a region recorded outbreak conditions for at least two years as regional outbreaks.

The regional records are each based on reconstructions completed at four to 24 individual sites covering a total sampled area of four to 120 hectares (Table 1). Within each study region, sites were dispersed over an average area of 372,082 hectares, based on rough estimates of the minimum bounding hulls enclosing all study sites. The authors of these studies reported successful matches between the reconstructed outbreak dates and observational records of twentieth century outbreaks in the general region of each study, suggesting that records based on only a few individual sites are often representative of WSB activity over larger areas. Furthermore, additional dendrochronological studies using sites not included in this analysis report generally similar outbreak patterns in northeastern Oregon [23] and central British Columbia [24] for the time period shared with the records I analyzed. These records should be treated as a conservative estimate of outbreak synchrony and activity because of the limited area sampled, but it appears likely that they are representative of outbreak histories for their respective regions.

**Table 1:** Outbreak record characteristics for regional records used in this study and the inter-regional record presented in this paper for the 1700–1990 common period. Regions are as follows: BC = central British Columbia, WA = northern Washington, MT = western Montana / eastern Idaho, ID = western Idaho / northeastern Oregon, OR = north-central Oregon, CO = southern Colorado, NM = northern New Mexico, All = synchronous outbreak record (years with outbreaks recorded in three or more regional records). Species are: PSME = Douglas-fir, ABGR = grand fir, ABCO = white fir. Area sampled is a very approximate estimate based on published site descriptions.

Region	# of outbreaks	Average duration	Average quiescent period	# of trees (sites) sampled	Host species sampled	Area sampled (hectares)
BC	8	12.6	28.3	264 (19)	PSME	<19
WA	14	8.1	13.1	69 (4)	PSME	<4
MT	14	12	8.8	90 (5)	PSME	<5
ID	11	10.3	15.7	63 (4)	PSME ABGR	<4
OR	15	7.8	11	70 (4)	PSME ABGR	<4
CO	8	28.7	9.7	316 (11)	PSME ABCO	<100
NM	11	13.8	13.5	1028 (24)	PSME ABCO	120
All	13	15.4	7.3			

The seven regional records used in this analysis span nearly the entire geographic range of WSB and each record contains between 300 and 385 years of outbreak history. All seven regional records share a common period covering 1700–1990, expressed as a binary record of outbreak and non-outbreak years. I defined synchronous periods as years in which at least three of the seven study regions experienced an outbreak for at least two consecutive years. Thus, years labeled as having synchronous outbreaks are periods in which outbreaks occurred in multiple distinct latitudinal and topographic settings separated by hundreds of km.

### Spatial patterns of outbreak synchrony

To assess widespread patterns of synchrony, I first quantified the degree to which outbreaks were occurring synchronously or independently in time and space. I used the one-dimensional multivariate Ripley’s K-function [25] to determine whether three types of discrete outbreak events (outbreak years, outbreak initiation dates, and outbreak cessation dates) occurred independently in time among regions. This technique quantifies the temporal clustering or dispersion between specific events at multiple locations. I used this technique to test for co-occurrence of outbreak event years among all seven study regions within increasingly long temporal search windows. K-values were calculated over the span of temporal windows varying from zero years (i.e., synchrony during the year of event) to 25 years. This technique checked whether, on average across the whole length of the common period, outbreak events in the different study regions occurred within one year of each other, within two years of each other, and so on up to a window of twenty-five years, more often than would be expected if the timing was random. I assessed the statistical significance of my results using 1,000 randomized simulations in which the records were shifted in a circular fashion by adding a random number of years to each record, rather than individually randomizing each year, in order to maintain the multi-decadal temporal patterns of outbreak dynamics in the randomized data. The statistical significance of synchrony was thereby tested against a null hypothesis in which WSB outbreaks do not occur synchronously across sites, yet the outbreak records retain the same temporal structure. I completed this analysis in K1D v1.2 [26] for the 1700 to 1990 common period. The K-function is somewhat difficult to interpret visually as synchrony and asynchrony are as their interpretation is dependent on the specific temporal window being analyzed. K-values can be transformed to the L-function, which standardizes the mean and variance across temporal windows and can thus be interpreted consistently across temporal windows. For ease of interpretation, I present the results as transformed to the L function [26] with an expectation of 0 for no dependence, values above zero indicating synchrony, and values below zero indicating asynchrony among regions.

I then assessed the relationship between the degree of similarity among outbreak histories in different regions and the geographic distance between those regions. Geographic distance between regions was calculated as the Euclidian distance between the approximate centroids of each pair of study regions. I also considered using distance between the closest edges of each pair of regions, rather than their centroids. The rank order of centroid-to-centroid and edge-to-edge distances are the same, and each pair of region edges is separated by at least 73 km. I therefore chose to use centroids for ease of calculation. To quantify the relationship between binary time series of outbreak status during the 1700 to 1990 common period, I calculated a similarity index using the Jaccard coefficient for each possible pair of regions as follows:

$$Outbreak\;Similarity\;Index=\frac{a+b}{a+b+c+d}$$

Where *a* is the number of years in which both regions recorded an outbreak, *b* is the number of years in which both regions recorded non-outbreak conditions, *c* is the number of years in which only the first region recorded an outbreak, and *d* is the number of years in which only the second region recorded an outbreak. This index represents the percent of years during the common period in which the two regions recorded the same outbreak status. I assessed the strength of synchrony as a function of geographic proximity by comparing the outbreak similarity index with the distance between region pairs. The strength of this relationship was tested using simple linear regression models and a Mantel test with significance assessed through 10,000 permutations implemented with the R package NCF [27]. The Mantel test controls for the dependence inherent in matrices of pairwise similarities when assessing the correlation between geographic distance and outbreak history similarity.

### Climate drivers of outbreak synchrony

I analyzed the relationship between synchronous outbreaks and climate variability over long time scales by comparing my record of synchronous outbreaks with records of moisture availability. I focused on moisture availability because it has been linked to WSB population fluctuations in previous analyses of both dendrochronological [15–17,21] and observational [28–31] outbreak records, as well in controlled experiments on WSB larvae [32–34]. To represent moisture availability, I used data from Cook et al.’s [35] gridded dendroclimatic reconstruction of the summer Palmer Drought Severity Index (PDSI). I chose to use PDSI as it provides an integrative index of moisture stress calculated using a combination of temperature, precipitation, and soil type data [36]. Both observational and dendroclimatic records of PDSI exhibit spatial variability in their correlation with seasonal precipitation anomalies [37]. PDSI in the Pacific Northwest tends to be most strongly correlated with summer precipitation, while PDSI in the American Southwest tends to be primarily correlated with winter precipitation [37]. In spite of these spatially variable seasonality signals, PDSI in both regions represents moisture availability during the summer [35,37]. Because summer moisture availability is the seasonal variable most strongly linked to WSB population fluctuations [28,29,31], the overall amount of moisture available to WSB and its host trees during the summer is likely more important than the seasonality of precipitation associated with that moisture. I obtained the PDSI records from the seven grid points closest to the seven WSB outbreak reconstructions (specifically, grid points 42, 43, 44, 56, 83, 118, and 133) for the 1700–1990 common period.

I quantified the spatial and temporal association between climate and periods of synchronous WSB outbreaks using four different approaches. First, I compared the spatial patterns of coherence between PDSI records with the spatial patterns of similarity in outbreak histories by calculating a similarity index based on the average annual absolute difference between PDSI values at each pair of regions. The PDSI similarity index was calculated for each possible pair of regions using the following equation:

$$Climate\;Similarity\;Index=-1*\frac{\sum_{t=1}^{n}\sqrt{{\left(x_{t}-y_{t}\right)}^{2}}}{n}$$

Where *x*_*t*_ is PDSI in one region for year *t*, and *y*_*t*_ is PDSI in a second region for year *t*, and *n* is the number of years in the common period. This similarity index represents the inverse of the average annual total Euclidean distance in climate space for each possible pair of regions. I quantified the relationship between this “climate space” similarity index and Euclidean geographic distance between the study region pairs using simple linear regression models and a Mantel test. Second, I tested whether outbreak histories were more similar among regions with more similar PDSI records using a Mantel test to compare climatic similarity and outbreak history similarity (measured using the Jaccard similarity index described above). To account for spatial autocorrelation, I also used a partial Mantel test to repeat this comparison while controlling for geographic distance. For all Mantel tests, statistical significance was assessed through 10,000 permutations implemented with the R package NCF [27].

Third, I used superposed epoch analysis to quantify climate anomalies associated with specific “events”, in this case the initiation and cessation of synchronous outbreaks. This technique creates a composite of the climatic conditions associated with events by averaging a climate variable for the years preceding and following each event [38]. In this case, the results tell us if conditions tended to be, on average, warmer/drier or cooler/wetter than the long-term average during years just before and after synchronous outbreak initiations and cessations. I conducted six separate analyses (both outbreak starts and ends for three regions) using this technique to look at start and end dates of synchronous outbreak periods. For these analyses, I created a single inter-regional index of PDSI by averaging the regional records, a “southwest” PDSI index by averaging the regional records from my two southwestern study areas (i.e., Colorado and New Mexico), and a “northwest” PDSI index by averaging the records from my five northwestern study areas. Outbreak initiation and cessation dates represent the start and end of periods in which outbreaks occurred synchronously across western North America, so the same set of event dates was compared with each regional index of PDSI. I computed composited climate anomalies over an 11-year window that includes the outbreak initiation or cessation date and the five years preceding and five years following that event. I examined longer temporal windows, but found no consistent patterns at longer lags. I assessed the statistical significance of the climate anomalies with bootstrapped confidence intervals derived from 1,000 Monte Carlo simulations [39]. Fourth, I tested for significant differences between the average inter-regional, southwest, and northwest PDSI index values during a) synchronous outbreak years and non-synchronous outbreak years, and b) the five years prior to each synchronous outbreak and all other years using a Welch t-test.

## Results

### Outbreak histories

The seven regions analyzed each experienced between eight and 15 outbreaks during the 1700–1990 common period (Table 1). The average duration of regional outbreaks ranged from 7.8 years to 28.7 years (mean = 13.3). The average quiescent period between outbreaks ranged from 8.8 to 28.3 years (mean = 14.3). There was no clear latitudinal gradient in these outbreak characteristics. For instance, the southern Colorado region had the longest average outbreak duration (28.7), while Oregon, the next region to the North, had the shortest average duration (7.8). The British Columbia region had a remarkably long average quiescent period (28.3 years), but this was largely the result of a single period of low WSB activity during the 1800s.

**Fig 2:**
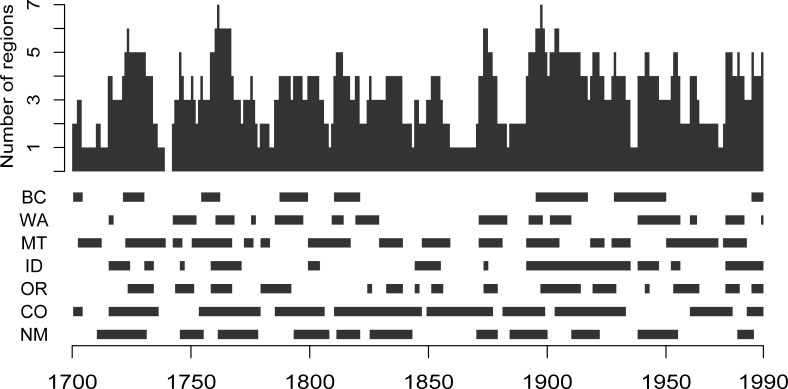
**Number of regional chronologies recording an outbreak (top) and reconstructed outbreak years for each region (bottom).** Regions are as follows: BC = central British Columbia, WA = northern Washington, MT = western Montana / eastern Idaho, ID = western Idaho / northeastern Oregon, OR = north-central Oregon, CO = southern Colorado, NM = northern New Mexico.

Visual assessment of the total number of regional reconstructions indicating an outbreak (Fig 2) reveals a moderate level of synchrony, with a clear pattern of temporal clustering of outbreaks. Lags of one to three years between the onset or cessation of defoliation and the beginning or end of reduced radial growth are common due to the host trees’ utilization of previously stored carbohydrates for radial growth and the time required to replenish photosynthetic tissue [23]. These lags could obscure underlying patterns of synchronous outbreaks if the lag times vary spatially and temporally. The results presented in this paper should thus be treated as a conservative estimate of the true level of synchrony.

I identified 13 distinct synchronous outbreaks (Table 2). Of these 13 synchronous outbreak periods, five involved all seven regions. By definition, at least three regions experienced outbreak conditions during each of these synchronous periods, and all but the two shortest synchronous periods included years in which at least four of the regions experienced outbreaks. While not every synchronous outbreak included all regions, it is notable that none of these synchronous outbreaks displayed a strong pattern of latitudinal clustering. In other words, none of the synchronous outbreaks I identified included only the southern half or only the northern half of the regions. The average duration of synchronous outbreaks was 15.4 years, but durations ranged from two to 44 years. The longest synchronous outbreak started in 1891 and lasted 44 years, with extensive outbreak periods occurring in all seven regions. The average quiescent period between synchronous outbreaks was 7.3 years.

**Table 2:** Synchronous outbreaks. Start year and end year of synchronous (i.e., outbreaks occurring simultaneously in three or more regions) period, duration (years) of synchronous outbreak, and individual regions recording an outbreak in at least one year during the period. Regions are as follows: BC = central British Columbia, WA = northern Washington, MT = western Montana / eastern Idaho, ID = western Idaho / northeastern Oregon, OR = north-central Oregon, CO = southern Colorado, NM = northern New Mexico. Note: the New Mexico record ends in 1990, in the midst of a widespread outbreak.

Start date	End date	Duration (years)	Regions						
1702	1703	2	BC		MT			CO	
1715	1733	19	BC	WA	MT	ID	OR	CO	NM
1743	1776	34	BC	WA	MT	ID	OR	CO	NM
1785	1805	21	BC	WA	MT	ID	OR	CO	NM
1810	1820	11	BC	WA	MT			CO	NM
1824	1838	15		WA	MT		OR	CO	NM
1844	1845	2				ID	OR	CO	
1849	1855	7			MT	ID	OR	CO	
1871	1878	8		WA	MT	ID	OR	CO	NM
1891	1934	44	BC	WA	MT	ID	OR	CO	NM
1938	1955	18	BC	WA	MT	ID	OR		NM
1960	1963	4		WA	MT		OR	CO	
1975	1990	16	BC	WA	MT	ID	OR	CO	NM

During the common period shared by all seven reconstructions (1700–1990) there were 199 years (68% of the common period) in which outbreaks were recorded simultaneously in three or more regions and 139 years (48% of the common period) in which outbreaks occurred simultaneously in four or more regions. Synchronous outbreak years became more common after 1890 (82% of years between 1890 and 1990 vs 63% of years between 1700 and 1889). There were only three years in which no region experienced outbreak conditions, and none of these years occurred during the 20^th^ century.

### Spatial patterns of outbreak synchrony

**Fig 3:**
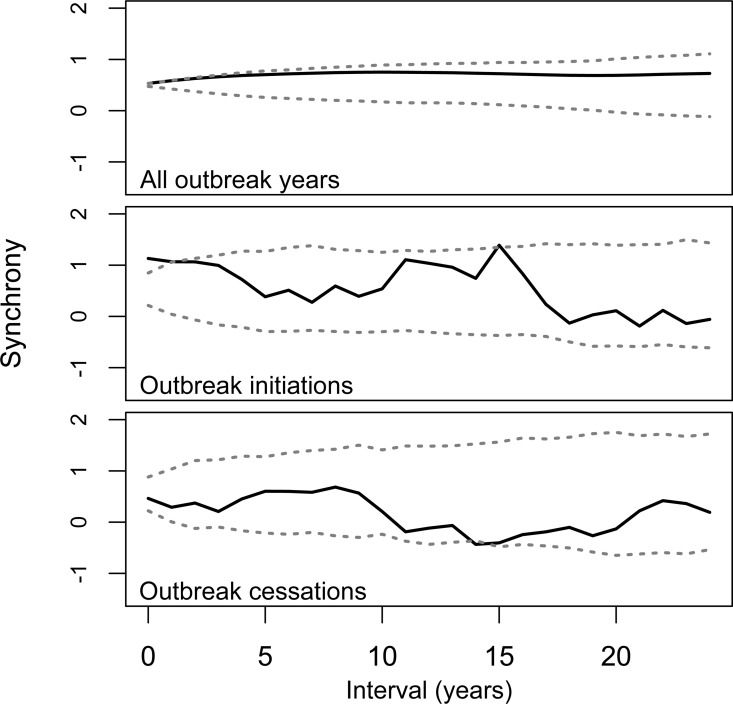
Modified multivariate Ripley’s K-function calculated for synchronous outbreak years, initiation dates, and cessation dates in all seven study regions over the 1700–1990 common period. Light grey lines represent a 95% confidence interval based on randomizing the outbreak records relative to each other.

The K-function calculated for outbreak initiation dates in all regions revealed statistically significant synchrony over temporal windows of zero, one, and 15 years (Fig 3). A similar analysis undertaken using outbreak cessation dates in all regions produced no statistically significant results, except a single year of asynchrony over a 14 year window. Given the lack of a consistent pattern of significant clustering over temporal windows greater than zero to one years, synchrony is likely focused on that temporal scale. When all outbreak years were assessed, low, but statistically significant synchrony was found over a zero-year window.

**Fig 4:**
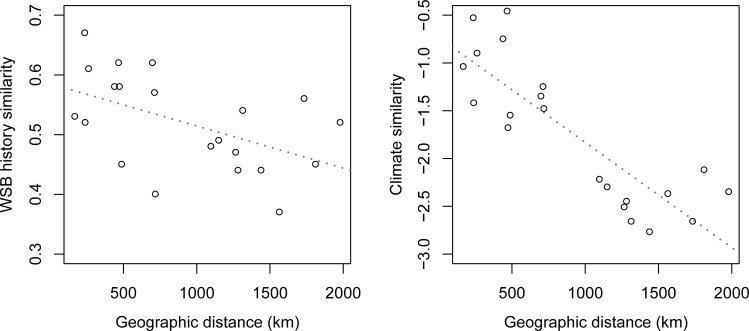
**Similarity among regional outbreak records (left) and regional drought indices (right) as a function of distance between study regions.**

Across the transect, the average percent of years in which any pair of study regions agreed on outbreak status was 52%. The level of agreement between study regions was moderately dependent on distance between regions (Fig 4). A simple linear regression line was able to predict 26% of the variance in inter-regional agreement based on inter-regional distance. A Mantel test comparing similarity of outbreak records and geographic distance showed a statistically significant decline in outbreak history similarity between region pairs with increasing distance (r = -0.51, p = 0.036). The relationship between inter-region distance and outbreak-record agreement was generally consistent across the transect. An exception to this pattern was seen in the records from British Columbia and eastern Idaho/western Montana, which had a much lower agreement than the linear regression model would predict.

### Climate drivers of outbreak synchrony

The spatial patterns of similarity between climate records (Fig 4) exhibited a distance decay trend similar to the spatial patterns of WSB synchrony. Similarity between pairs of PDSI records steadily declined with increasing distance and this relationship was considerably stronger than the distance decay pattern seen in outbreak records. A simple linear regression model was able to explain 72% of the variance in the correlation between study regions as a function of inter-regional distance. This pattern of spatial autocorrelation was statistically significant according to a Mantel test (r = -0.85, p = 0.008). The bimodal clustering of pairwise similarity values apparent in Fig 4 follows the “dipole” climatological pattern known to exist wherein Pacific Northwest and American Southwest often experience opposite precipitation anomalies [40,41]. Interestingly, this spatial clustering pattern is much weaker in the outbreak history similarity index.

Similarity of outbreak histories and climatic similarity between region pairs were significantly positively correlated according to a Mantel test (r = 0.63, p = 0.012). The strong spatial autocorrelation apparent in both outbreak and climate records could lead to spurious correlations between the two variables. I therefore used a partial Mantel test in which geographic distance was effectively held constant while climate and outbreak history similarities were compared. This was done through a partial regression, wherein the final results represent the correlation between one variable and the residuals of another after the variability explained by geographic distance has been removed. Once spatial autocorrelation was controlled for, there was still a statistically significant relationship between outbreak history similarity and climatic similarity (r = 0.43, p = 0.044). However, when climatic similarity was held constant, the relationship between outbreak history similarity and geographic distance was no longer statistically significant (r = 0.06, p = 0.432).

**Fig 5:**
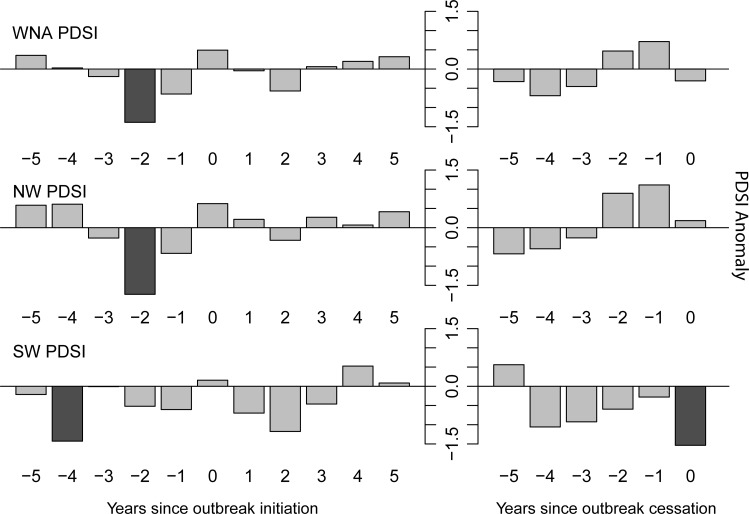
**Superposed epoch analysis results for Palmer Drought Severity Index values during years of initiation (left column) and cessation (right column) of synchronous western spruce budworm outbreaks.** Results are shown for PDSI averaged across all regions (top row), PDSI averaged across the five Pacific Northwest regions (middle row), and PDSI averaged across the two Southwest regions (bottom row). Descending bars represent warmer/drier than average conditions, ascending bars represent cooler/wetter than average conditions. Dark grey shading indicates statistically significant (at the 95% confidence interval) anomalies.

The superposed epoch analysis showed that average PDSI across the study area was statistically significantly lower than average two years before the initiation of synchronous outbreaks (Fig 5). When examined separately, PDSI anomalies in the northwest and southwest were both below average for at least three of the five years before synchronous outbreak initiation, but the exact timing of the strongest negative anomalies varied among regions. PDSI averaged across the five northwestern study areas was significantly below average two years before synchronous outbreak initiations, while PDSI averaged across the two southwestern study areas was significantly below average four years prior to the same initiation dates. I found no consistent relationship between outbreak cessation and PDSI. I report only results for the year of cessation and preceding years, as subsequent years’ climate could not affect prior population collapse. No significant values were apparent for the years leading up to outbreak collapse (i.e., the years in which climate could induce population collapse).

PDSI averaged across all study regions over the five years prior to start dates of synchronous outbreaks was statistically significantly (Welch t-test, t = -2.35, p = 0.021) warmer/drier (average PDSI = -0.43 ± 1.56) than other years (average PDSI = 0.10 ± 1.64). This pattern held true when analyzing only climate from the northwest, with a statistically significant (Welch t-test, t = -2.09, p = 0.039) trend for warmer/drier (average PDSI = -0.39 ± 1.84) conditions during the five years prior to outbreak initiation compared to all other years (average PDSI = 0.16 ± 1.89). When analyzing only climate from the southwest, the results were not statistically significant (Welch t-test, t = -1.53, p = 0.130), but exhibited a similar pattern of warmer/drier (average PDSI = -0.61 ± 2.25) conditions in the five years prior to outbreak initiation than in other years (average PDSI = -0.13 ± 2.04). Average PDSI across the study region was statistically significantly (Welch t-test, t = 2.635, p = 0.009) cooler/wetter (average PDSI = 0.15 ± 1.65) during synchronous outbreak years than during non-outbreak years (average PDSI = -0.35 ± 1.55). Average PDSI across the five northwestern sites was statistically significantly (Welch t-test, t = 2.21, p = 0.028) higher during synchronous outbreak years (average PDSI = 0.21 ± 1.90) than during non-outbreak years (average PDSI = -0.31 ± 1.82). Average PDSI across the two southwestern sites was also statistically significantly (Welch t-test, t = 2.58, p = 0.011) higher during synchronous outbreak years (average PDSI = -0.02 ± 2.01) than during non-outbreak years (average PDSI = -0.70 ± 2.08).

## Discussion

A very widespread WSB outbreak occurred during the late 20th century, with more than five million hectares affected during 1985 alone [42]. This extensive disturbance drew attention to WSB dynamics and raised questions about whether such large outbreaks were within the natural range of variability for this insect. Analyzing dendrochronological records from across most of the latitudinal range of the WSB clearly shows that WSB outbreaks have occurred synchronously across large areas of western North America multiple times over the last three centuries. Synchronous outbreaks appear to be a normal part of this species’ population dynamics. However, these same records show an increase in the frequency of synchronous outbreak years after the late 19^th^ century, perhaps in response to land use changes that have increased the density of the WSB’s host species [43,44]. These dendrochronological records offer valuable insight into temporal changes in WSB population dynamics over multiple centuries, but do not have the spatial coverage needed to determine whether the area of outbreaks has increased in response to the increase in the area occupied by dense forests of their primary host. The limited area covered by each regional record means that my results should be treated as a conservative estimate of synchrony, as some synchronous outbreaks may have been missed if they did not occur in these specific regions. Future research is warranted to explore broad-scale spatial, as well as temporal, changes in WSB outbreak dynamics.

Outbreak years and outbreak initiation dates in the seven regions I analyzed were significantly clustered in time. Both events were clustered over temporal windows of zero to one years, indicating a narrow temporal window of synchrony. Synchrony over longer temporal windows was generally not statistically significant, but two instances of significant synchrony of outbreak initiations and asynchrony of outbreak cessations at temporal windows of 14–15 years suggests the possibility of weaker decadal-scale patterns of temporal clustering. Overall, these patterns indicate a broad scale synchronization of population trends interacting with local site conditions to determine the exact date at which WSB density crosses the threshold into outbreak conditions. WSB outbreaks have occurred synchronously across regions up to 2,000 km apart, though synchrony declined with increasing geographic distance between regions. This pattern of synchronous population dynamics has been observed in many plant and animal species, yet it is often extremely difficult to isolate the actual underlying causal mechanism or mechanisms for this phenomenon [9,10]. In this paper, I seek to identify whether climate may be a primary causal factor behind the very widespread synchrony I identified, but quantifying the exact contribution of different factors is not possible given the likelihood of complex interactive effects [9,10]. Several different mechanisms have been proposed to explain the observed patterns of synchrony in different species.

One possible synchronizing mechanism is dispersal. For mobile species, the dispersal of individuals from areas of high population density to areas of low density may act to synchronize population fluctuations. Dispersal may be an effective synchronizing mechanism for relatively unstable populations [45]. Dispersal’s efficacy as a synchronizing mechanism may be enhanced by, or even dependent on, the presence of predators, due to the ability of predation to increase cyclicity in a prey’s populations [46]. The limited dispersal range of many species means dispersal may only be an important synchronizing factor at relatively small spatial scales for these species, and is therefore generally considered unable to explain the observed synchrony at larger spatial scales [1,7,47]. Reports of similar levels of spatial synchrony in multiple insect species with highly variable dispersal abilities [1] strongly suggest that synchrony of these species must be driven by large-scale exogenous factors rather than dispersal. The dispersal capabilities of the WSB are not well known [31,48]. Female moths of a similar species—the eastern spruce budworm (*Choristoneura fumiferana*)—are able to migrate over 100 km in a season [49], but the dispersal ability of the WSB is likely lower than that of the eastern spruce budworm [48]. Lower dispersal ability is a plausible explanation for the much higher intra-specific genetic diversity, and thus presumably lower inter-population gene flow, of the WSB when compared with the eastern spruce budworm [50]. Genetic analyses of WSB have shown considerable genetic exchange among sites less than approximately 350 km apart [48]. These results imply that dispersal may occur regularly among populations within relatively small areas, but is far less common over larger spatial scales [48]. Annual dispersal on the order of 100 km seems an unlikely, though not theoretically impossible [51], explanation for the synchronous population fluctuations I identified at distances of up to 2,000 km.

A second commonly invoked causal mechanism for synchrony is the influence of regional stochasticity. This hypothesis, commonly known as the Moran effect [52], proposes that variability of spatially autocorrelated exogenous factors, particularly those related to climate conditions, could lead to synchronization of disjunct populations. According to this hypothesis, population fluctuations need not continuously track climatic fluctuations to be synchronized. Rather, if certain thresholds of the exogenous factor are crossed, this can push multiple disjunct populations into a positive or negative growth phase. The influence of climatic variability on a synchronized species may be indirect, as climatic synchronization of species can propagate through multiple trophic levels [6] and can also alter the rate and spatial patterns of dispersal [53,54]. Abiotic exogenous forcing mechanisms commonly called upon to explain observed patterns of synchrony include climate variables known to be correlated over large spatial scales [11], such as monthly temperature or precipitation, and annual indices of broad-scale atmospheric oscillations [1,2,5].

Strictly speaking, the Moran theorem requires that the populations in question have homogeneous, linear density dependence structures. The range of outbreak durations and time between outbreaks seen in the records I analyzed suggest locally varying population dynamics. Broadly similar, but not identical, periodicities have been reported for WSB outbreak records in different regions [20]. Differences in non-climatic conditions, such as predator characteristics, habitat quality, disturbance regimes, or land management practices, may be responsible for this geographic variability [1,45,55]. Local variations in WSB population dynamics violate a key assumption behind the classic Moran theorem, which may help to explain why many, but not all, outbreaks in the records I analyzed were synchronous across western North America. Additional non-synchronous variability may come from local climate differences. Precipitation anomalies are known to frequently vary in opposition in the Pacific Northwest and American Southwest [40,41]. This dipole pattern has been linked to spatially variable hydroclimatic responses to El Niño-Southern Oscillation and Pacific Decadal Oscillation teleconnections [40,41]. Extended periods of opposing precipitation anomalies may play a role in producing periods of non-synchronous outbreak conditions. Synchronous outbreak initiations may be more likely in periods with reduced expression of the dipole pattern. In spite of the potential desynchronizing effect of these local variations, climatic patterns appear to be closely linked to sub-continental scales of WSB outbreak synchrony.

A third possible synchronizing mechanism involves trophic interactions whereby predators could induce synchrony in their prey by responding to changes in the density of their prey numerically by migrating to areas of high prey density [56] or functionally by changing the composition of their diet in response to increased availability of a prey species [57]. Birds, ants, and other predators can play an important role in regulating WSB populations, especially during non-outbreak conditions, at the scale of forest stands [31]. Further research is needed to fully understand the potential effects of predators on WSB population fluctuations. However, based on our current knowledge, WSB’s predators do not appear to have the strong, responsive control over high density populations required to be a primary factor in synchrony of outbreaks [18].

My results support the hypothesis that broad-scale climate fluctuations play a key role in synchronizing WSB outbreaks across western North America. I found a close match in the spatial patterns of coherence among PDSI records and synchrony of WSB outbreaks. Even after controlling for spatial autocorrelation, climatic similarity and outbreak history similarity remained significantly positively correlated. Conversely, when climatic similarity was held constant, the relationship between outbreak history similarity and geographic distance vanished. Given that dispersal is inherently distance dependent, if dispersal of female WSB moths was the sole or primary factor responsible for synchronizing disjunct populations, we would expect to see a robust relationship between outbreak synchrony and geographic distance, even when climatic similarity is controlled for. These results, in conjunction with the significant climate anomalies associated with synchronous outbreaks and our existing knowledge of WSB’s spatially limited dispersal habits, strongly imply that climate, more than dispersal, synchronizes WSB outbreaks at sub-continental scales.

Looking closer at climate-synchrony associations, I found a statistically significant relationship between summer moisture availability and the initiation of synchronous outbreaks, as shown in the t-test and superposed epoch analysis results. These results indicate that fluctuations in summer moisture availability occurring over large areas of western North America have the potential to generate the observed patterns of spatial synchrony in these outbreak records. Moisture availability has been identified as a key driver of WSB population dynamics in stand-level analyses of both dendrochronological reconstructions [15–17,21] and observational records of twentieth century outbreaks [28,30,31]. Low moisture levels can increase survival and growth rates of WSB larvae via changes in the foliar chemistry of host trees [31–33]. However, prolonged moisture stress may have negative consequences for WSB [58,59], perhaps due to reductions in needle production that occur during droughts [60]. Intermittent moisture stress, as indicated by the reversal from below average to average or above average moisture availability in the t-test and superposed epoch analysis results presented here, has been suggested to provide the ideal conditions for outbreaks of many phytophagous insect species [58,59]. This pattern of a reversal from below average to above average PDSI around the start of synchronous outbreaks was apparent in both the American Southwest and the Pacific Northwest climate records. These sub-regional analyses were not identical, but showed a remarkable level of agreement, especially considering the dipole pattern of opposing fluctuations in precipitation frequently observed between these two regions [41]. Given the lags of one to three years previously reported between the start of outbreak-level WSB defoliation and the onset of radial growth reductions [23], the exact timing of climate anomalies relative to outbreak initiation should be interpreted with some caution. Climate anomalies at one to three years prior to outbreak initiation in the tree-ring record may actually be the climate anomalies associated with the start of heavy defoliation. These results focus only on synchronous outbreaks; outbreaks restricted to a single region may be associated with different climate conditions. Regional variability in climate-outbreak associations is an interesting topic that deserves further future research.

Outbreak cessation dates were not significantly clustered in time and were not consistently associated with any particular set of climatic conditions, which implicates local conditions related to variables such food availability and predators as the main driver of WSB population decline at the end of outbreaks. Synchrony of WSB outbreaks thus appears to be a result of synchronization of the timing of population increases, rather than synchronization of population collapse timing.

Synchronization of populations is a scale-dependent phenomenon, and different causal mechanisms may dominate at different spatial scales [1,47]. The WSB and many other species are probably synchronized through a combination of multiple mechanisms, with the relative importance of different mechanisms depending on the spatial scale of analysis and likely varying over space and time. Additionally, complex interactive effects can occur between causal mechanisms [9,10] and the distinction between causal mechanisms may be overly simplistic, as climate can alter dispersal patterns [53,54] and indirectly synchronize multiple species through effects that propagate between trophic levels [6]. While trophic interactions and dispersal undoubtedly play an important role in synchronizing some species at specific spatial scales, the large-scale synchrony of WSB populations that I detected appears more likely to be related to a mechanism operating at a similarly large scale. The significant matches in both the temporal and spatial patterns of climate and outbreak histories that I found suggest a robust role of climate in synchronizing distant WSB populations. Exogenous forcing, in the form of climate variability and discrete weather events, seems to be the mechanism best able to explain the observed large-scale synchrony of the WSB. However, the lack of a perfect match in the distance-dependent decay of climatic similarity and outbreak history similarity suggests that interactive effects, perhaps related to dispersal and habitat characteristics, also shape synchrony patterns.

## Conclusions

Analyses of synchronous population fluctuations are typically hampered by short observational records. In this study, I compiled multi-century dendrochronological records of WSB outbreaks to overcome this limitation. My analysis revealed statistically significant synchrony among regional WSB outbreak records across much of the species’ range. Numerous synchronous outbreaks occurred over the last three centuries and outbreak initiations were statistically significantly clustered in time. Synchronous WSB outbreaks are within the natural range of variability for this disturbance type, but the frequency of widespread synchronous outbreak years was higher in the 20th century than in prior centuries, likely due to land use induced changes in the availability of host trees. The sub-continental scale of synchrony identified through my analyses suggests that exogenous forcing plays a critical role in the synchronization of WSB population across much of western North America. The strong match in the spatial patterns of coherence among regional climate records and synchrony among outbreak records that I found supports the hypothesis that synchrony is linked to broad-scale climate variability. This is further corroborated by the significant associations that I identified between specific climate patterns and synchronous outbreak events. Synchronous outbreaks tended to begin during periods of average or above-average moisture availability preceded by one to five years of below average moisture availability. Based on these results, we can expect future synchronous outbreak patterns to be shaped by projected changes in climate, especially in terms of the frequency and duration of droughts, in conjunction with land use changes and the occurrence of other disturbances that can alter the density and contiguity of host species forests. My results indicate that spatiotemporal patterns of moisture availability have played a key role in synchronizing WSB population fluctuations in disjunct forests across western North America for at least the last three centuries and will continue to do so in the future.
